
JOURNAL INFORMATION
==============================
NLM Title Abbreviation: ScientificWorldJournal
Journal ID: ScientificWorldJournal
NLM Journal ID: 101131163
Journal ID: TSWJ

The Scientific World Journal

ISSN: 2356-6140
EISSN: 1537-744X
Publisher: Wiley

ARTICLE INFORMATION
==============================
PMCID: PMC6084219
PMID: 30147487
DOI: 10.1100/tsw.2001.142
Article ID: 953636
Article version: 1
Subjects: Short Report

Ischemia Elicits a Coordinated Expression of Apoptotic Proteins in Mouse Myocardium

Lyn Deborah 1 *
Lui Xiaowie 1
Bennett Nicole A. 1
Bao Shaojia 2
Emmett Nerimiah L. 2
1 Departments of Microbiology, Biochemistry & Immunology, Morehouse School of Medicine, Atlanta, GA 30310, USA
2 Department of Physiology, Morehouse School of Medicine, Atlanta, GA 30310, USA
*Deborah Lyn: lyn@msm.edu
Publication date: 2001
Electronic publication date: 2001 Oct 11
Volume: 1
First page: 39
Last page: 39
Copyright: Copyright © 2001 Deborah Lyn et al.
Copyright year: 2001
License: This is an open access article distributed under the Creative Commons Attribution License, which permits unrestricted use, distribution, and reproduction in any medium, provided the original work is properly cited.
License URL: https://creativecommons.org/licenses/by/3.0/

Funding: National Institutes of Health GM-08248 National Institutes of Health RR-03034

==============================
Miami *Nature Biotechnology* Short Reports
*TheScientificWorld* (2001) 1(S3), 39SR
ISSN 1532-2246; DOI 10.1100/tsw.2001.142

### ISCHEMIA ELICITS A COORDINATED EXPRESSION OF APOPTOTIC PROTEINS IN MOUSE MYOCARDIUM

Deborah Lyn[1,*], Xiaowei Lui[1], Nicole A. Bennett[1], Shaojia Bao[2], Nerimiah L. Emmett[2]

Departments of [1]Microbiology, Biochemistry & Immunology, [2]Physiology,
Morehouse School of Medicine, Atlanta, GA 30310, USA
* lyn@msm.edu

**INTRODUCTION.** Cardiac ischemia has been associated with distinct structural and intersecting cellular pathways that contribute to the ventricular remodeling process. Damaging effects of ischemic injury may trigger the apoptotic cascade while a counterbalancing stimulation of cell survival pathways may limit cell death to the infarcted region. The ARC (apoptosis repressor with caspase recruitment domain) protein is predominantly expressed in cardiac and skeletal muscle, and functions as an inhibitor of apoptosis via selective interactions with caspases (1). An evaluation of the role that ARC plays during ischemia/hypoxia remains sparse. Protein expression of ARC was examined and compared to other prominent apoptotic molecules using an *in vivo* murine model of ischemia.

**METHODS.** Cardiac ischemia was induced using an established microsurgical murine model involving constriction of the left anterior coronary artery (2). Specific primers to *Bcl-xL* and *Bax* mouse genes were obtained from Clontech and RT-PCR assays conducted as recommended. Myocardial protein levels were monitored by western blot analysis from at least 3 separate animals. Antibodies were obtained from the following sources: ARC, p53 (Oncogene Research Products); BID, Bcl-xL, Bax (BD Sciences); phosphospecific Akt (Cell Signaling Technology).

**RESULTS AND DISCUSSION.** Initial experiments using a cDNA expression array strategy showed that transcription levels of anti-apoptotic molecules such as $Bcl\text{-}x_L$, *BAG-1* (bcl-2 binding protein) and *Akt* were unaltered after 24-hr ischemia (2). We found an increase in *Bax* to *Bcl-xL* transcript levels after 3 to 14 days of ischemia relative to sham-operated heart tissue, with similar findings after examination of protein expression (Table 1).

In this study, a reduction in ARC expression (30-60%) was observed after 24-hr of ischemia without alterations in protein levels at longer periods. These data support other *in vitro* studies that show lower ARC protein levels after hypoxia in an embryonic derived heart cell line (3). Truncated levels of BID (t-BID) were seen at all time points of ischemia which suggest that factors other than ARC may interact with caspase-8. The amounts of p53 (Bax gene transcription factor) protein remained unaltered despite increases in Bax expression. Therefore, cardiac ischemia may occur via a p53-independent pathway that differs from tumor models of apoptosis. At extended periods of ischemia (after 14 days), increases in Bcl-xL and the phosphorylated form of Akt indicated that anti-apoptotic factors limit myocardial ischemic injury.

Table 1. Summary of Relative changes in Protein Levels

| Days After Ischemia | | | | | |
|---|---|---|---|---|---|
| | 1 | 3 | 7 | 14 | 142 |
| ARC | ↓ | (-) | (-) | (-) | (-) |
| Phospho-Akt | ↑ | (-) | (-) | (-) | ↑ |
| Bcl-xL | (-) | (-) | (-) | ↑ | ↑ |
| Bax | (-) | ↑ | ↑ | ↑ | (-) |
| t-BID | ↑ | ↑ | ↑ | ↑ | ↑ |
| p53 | (-) | (-) | (-) | (-) | ND |

(-), no change; ↓, decrease or ↑, increase in protein levels from ventricle tissue relative to sham-operated after normalizing to tubulin. (ND) Not determined.

In summary, a 24-hr time interval was associated with loss of protective mechanisms against ischemia-induced apoptosis.

**ACKNOWLEDGEMENTS.** Support was provided by NIH Grants GM-08248 and RR-03034.

**REFERENCES.**

1. Koseki, T., Inohara, N., Chen, S., and Núñez, G. (1998) Proc. Natl. Acad. Sci. U S A 95, 6156-5160
2. Lyn, D., Liu, X., Bennett, N.A., and Emmett, N.L. (2000) Physiol. Genomics 2, 93-100
3. Ekhterae, D., Lin, Z., Lundberg, M.S., Crow, M.T., Brosius, F.C., and Núñez, G. (1999) Circ. Res. 85, e70-e77
